# Diet supplemented with fermented onion improves growth performance, health condition, meat quality, and modifies rumen metabolite profiles in Liangshan black sheep

**DOI:** 10.3389/fvets.2025.1695023

**Published:** 2025-10-29

**Authors:** Shengwang Jiang, Chaoyun Yang, Chen Ji, Chao Li, Gang Lv, Hongwei Gao, Wei Zhang, Yi Zhang, Zengwen Huang

**Affiliations:** ^1^College of Animal Science, Xichang University, Xichang, Sichuan, China; ^2^Key Laboratory of Local Characteristic Goat at Xichang University of Sichuan Province, Xichang, Sichuan, China; ^3^College of Agricultural Science, Xichang University, Xichang, Sichuan, China; ^4^Xinjiang Taikun Group Co., Ltd., Changji, Xinjiang, China; ^5^College of Animal Science and Technology, Xinjiang Agricultural Vocational and Technical University, Changji, Xinjiang, China

**Keywords:** fermented onion, Liangshan black sheep, growth performance, rumen metabolite profiles, meat quality

## Abstract

This study aims to investigate the effect of fermented onion on Liangshan black sheep’s growth performance, health, meat quality, and rumen metabolite profiles. A total of 80 four-month-old female Liangshan black sheep were randomly divided into four groups of five replicate pens (four sheep per pen). Sheep were fed a basal diet supplemented with 0 (control), 10, 20% or 30% fermented onion. Compared to that of the control group, dietary supplementation with 20% fermented onion improved final body weight, ADG and ADFI; enhanced GPT and GOT activities and increased IgA, IgG, IgM, C3, and C4 levels; increased the levels of IL-4, IL-10, TGF-*β* and decreased the levels of IL-1β, IL-6, TNF-*α*, IFN-*γ*; decreased MDA level and increased SOD, GST, CAT and GSH-Px activities; decrease the pH, L^*^ value, b^*^ value and shear force; increase the a^*^ value and the content s of protein and fat; increase the expression levels of FN1, TGFβ1, Myf5, FAS, PPARγ and FABP4; decrease the expression levels of CPT1A and LPL. Metabolomic analysis revealed that 20% fermented onion supplementation significantly modified the metabolite profiles in the rumen liquid, with 44 downregulated metabolites and four upregulated metabolites mainly enriched in purine metabolism, microbial metabolism, cutin, and suberine biosynthesis pathways. Data from our study suggest that supplementation of fermented onion to the basal diet at 20% (w/w) can be used safely to increase meat yield and to improve meat quality in the sheep industry.

## 1 Introduction

Xichang city has a mild climate, fertile land, and a convenient irrigation system. Abundant light, warm weather, and land resources create favorable conditions for onion cultivation. Xichang onion has the characteristics of fresh color with tight structure and rich nutrients, crisp and sweet taste with fragrant smell and tenderness, large fruit that is easy to store, and resistant to transportation. It contains 17 amino acids, vitamin C, trace element selenium, etc., plus garlic, prostaglandin A, and quercetin, which are unique to onion. Additionally, Xichang onion contains a variety of substances that are bactericidal and anti-inflammatory, anti-aging, lowering blood pressure, preventing cancer, improving immunity, and preventing osteoporosis. Due to its unique properties, the Xichang onion is greatly favored by consumers. In 2010, Xichang onion was certified as a national geographical indication product by AQSIQ (General Administration of Quality Supervision, Inspection and Quarantine of the People’s Republic of China).

The onion planting area in Xichang is about 5,000 hectares, and the total output exceeds 250,000 tons. Xichang is regarded as one of the main onion planting areas in China. The production of onions and their by-products (such as onion stems and leaves) has steadily increased yearly, resulting in high pressure on onion sales and sharp price fluctuations. In addition, a large amount of onion waste is generated every year (1). Thus, it is an urgent and critical issue to explore the full utilization of onions and their by-products (2).

On July 1, 2020, the document No. 194 from the Ministry of Agriculture and Rural Affairs of the People’s Republic of China was implemented, marking China official entry into the era of “feed prohibition” (no antibiotic is allowed in animal feeds). This makes animal husbandry enterprises face a major challenge of frequent disease. So, to promote the green and sustainable development of the livestock and poultry industry, it is urgent to find high-quality, efficient, green, and safe new feed additives with antibacterial activities. Studies have shown that onions contain a large number of natural antibiotics, which have strong bactericidal activities that can kill or inhibit *Staphylococcus aureus*, *Streptococcus*, diphtheria, dysentery, tuberculosis, and *Escherichia coli* (3). In addition, onions and their extracts, when used as feed additives, play an important role in improving the production performance, immunity, nutritional digestibility, and meat quality of livestock and poultry (4–8). Onions are rich in carbohydrates, vitamin C, and trace elements such as potassium, calcium, and phosphorus. Studies have shown that the bioactive substances in onion extract, such as sulfur-containing compounds, flavonoids, polysaccharides, and saponins, can improve the body weight and feed conversion efficiency of broilers. Therefore, onions have potential antibacterial and growth-promoting value (9, 10). Since fresh onions contain high levels of sulfur compounds (such as dipropyl disulfide, dipropyl trisulfide, etc.) in vacuoles, these sulfur compounds are released and contacted with onion enzymes, resulting in the generation of volatile substances with a pungent smell once the chopped onions are eaten and chewed by animals. These volatile sulfur compounds can irritate the eyes and respiratory nerves, causing reactions such as tears and coughing, thereby making unfermented onions much less palatable as a feed material for animals. By undergoing the natural fermentation after adding sugar and salt to chopped onions, most sulfides in fresh onions will be converted into sweet substances, reducing the spiciness and improving the onion’s palatability.

Liangshan black sheep is one of the top 10 excellent germplasm resources of livestock and poultry in China. Due to its strong and tall physique, symmetrical structure, high meat quality, genetic stability and strong adaptability, easy feeding, and the combined use of meat and hair characteristics, it has been certified as a national geographic indication product in 2020 (11, 12).

However, the poor growth performance seriously restricted the development of the Liangshan black sheep industry. Because the Liangshan black sheep are mainly raised by grazing and supplementary feeding, thus, the growth rate is slow and the breeding cycle is long. The rumen, as the central site of microbial fermentation, is the critical interface where diet influences host metabolism. While the positive outcomes are clear, how fermented onion precisely alters the metabolic network within the rumen to fuel superior growth and health is not understood. Therefore, this study was designed to make full use of Xichang onion and Liangshan black sheep to investigate the effect of adding fermented onion to the basic diet on Liangshan black sheep’s growth performance, health, meat quality, and rumen metabolite profiles. By generating new data and understanding the molecular mechanism of fermented onion, we aim to make full use of onion resources in Xichang and promote the development of the Liangshan black sheep industry.

## 2 Materials and methods

### 2.1 Fermented onion preparation

The onion was peeled, with heads and roots being removed, and the remaining portion was chopped into pieces of approximately 1.5 cm × 1.5 cm in size. Subsequently, 5% sugar and 1% salt were added to the chopped onion pieces, and the mixture was agitated at 20–25 °C until the onion juice was released. The agitation took about 2 h. Once the agitation was completed, the mixture was then transferred to a food-grade fermentation container and placed in a 15–20 °C environment. The container was incubated at room temperature for a period of 25 days, during which time the fermentation process took place in an anaerobic environment. A successfully fermented onion had the following characteristics: soft in texture, exuding a sour smell and a robust aroma, and no pungent odor or evidence of mildew.

### 2.2 Ethics statement

The experimental protocol and procedures were approved by the Experimental Animal Welfare and Ethics Committee of Xichang University (permit number for conducting animal experiments: No. xcc2025001). All animal treatments and experiments were performed according to the recommendations described in the guidelines for ethical review of animal welfare in the national standards of the People’s Republic of China (151).

### 2.3 Experimental design and animal feeding management

A single-factor test design was used for our experiment in this study. A total of 80 four-month-old female Liangshan black sheep (initial body weight: 23.40 ± 0.36 kg) in good health were selected and randomly divided into four groups, with a total of 20 animals per group and five replicates per group. Each replicate had four sheep in a pen. The control group (Con) was fed with the basic diet (Xinjiang Taikun Group Co., Ltd.), while the experimental groups were fed with the basic diet supplemented with 10% (Trt1), 20% (Trt2), and 30% (Trt3) fermented onion, respectively. All diets were isonitrogenic and isoenergetic, and the ingredient composition and nutrient levels (measured values) of diets are listed in Table 1. The percent of fermented onion was calculated by weight (dry matter). For example, 2 kg of fermented onion added to 8 kg of the basal diet resulted in a diet with 20% fermented onion. The basal diet was formulated to meet sheep nutrient requirements during the experimental period. The pre-experiment lasted for 7 days, and the experiment lasted for 70 days. All sheep were fed twice a day (08:30 and 18:30) according to the daily management methods of the sheep farm and had ad libitum access to food and water. Sheep pens were kept clean and dry, and disinfected regularly.

**Table 1:** Ingredient composition (%) and analytical composition in each diet.

Items	Groups			
	Con	Trt1	Trt2	Trt3
Ingredient composition (%)				
Corn	33.00	29.70	26.40	23.10
Cotton meal (42% CP)	10.00	9.00	8.00	7.00
Spray corn husks	10.00	9.00	8.00	7.00
Wheat bran	10.00	9.00	8.00	7.00
Extruded soybean	2.00	1.80	1.60	1.40
Corn DDGS	5.00	4.50	4.00	3.50
Rice bran	10.00	9.00	8.00	7.00
Corn stalks	10.00	9.00	8.00	7.00
Vitamin-mineral premix	10.00	9.00	8.00	7.00
Fermented onion	0.00	10.00	20.00	30.00
Total	100	100	100	100
Analytical composition in diet				
Net energy (NE, kcal/kg)	2850.00	2851.00	2852.00	2853.00
Dry matter (DM, %)	88.69	88.82	88.95	89.08
Crude protein (CP, %)	14.42	14.23	14.04	13.84
Neutral detergent fiber (NDF, %)	26.00	25.80	25.60	25.40
Acid detergent fiber (ADF, %)	16.20	16.33	16.46	16.59
Crude fiber (CF, %)	11.40	11.51	11.62	11.73
Crude ash (CA, %)	11.20	11.08	10.96	10.84
Calcium (Ca, %)	0.65	0.65	0.64	0.64
Total phosphorus (TP, %)	0.55	0.54	0.53	0.52
Sodium chloride (NaCl, %)	0.33	0.34	0.34	0.35

### 2.4 Growth performance

Each sheep was weighed before the experiment began (initial weight) and after the experiment was completed (final weight). All sheep were fasted for 12 h before weighing. Simultaneously, the feed intake and leftover feed of each pen were recorded daily. The average daily gain (ADG) in body weight, average daily feed intake (ADFI), and feed conversion ratio (FCR) were calculated using the daily data collected during the study period.

### 2.5 Blood sample collection and analysis

At the end of feeding experiment, one sheep in each replicate was randomly selected and euthanized, followed by bloodletting and slaughter. Blood samples (5 mL) were collected in 10 mL anticoagulant-free vacutainer tubes and immediately centrifuged at 3,000 × g for 10 min at 4 °C to obtain serum. Serum samples were stored at −20 °C until further analysis (serum biochemical and immunological parameters, serum inflammatory factors, and antioxidant capacity).

#### 2.5.1 Serum biochemical parameters

The contents of total protein (TP), albumin (ALB), globulin (GLB), glutamic pyruvic transaminase (GPT), and glutamic oxaloacetic transaminase (GOT) in serum were determined using an automatic biochemical analyzer (BK-280, Biobase, China).

#### 2.5.2 Serum immunological parameters

The levels of serum immunoglobulin A (IgA), immunoglobulin G (IgG), immunoglobulin M (IgM), complement 3 (C3), and complement 4 (C4) were determined using enzyme-linked immunosorbent assay (ELISA) kits according to the manufacturer’s instructions (Jiangsu Enzyme Exemption Industry Co., Ltd., China). All samples were measured in quadruplicate.

#### 2.5.3 Serum inflammatory factors

The levels of interleukin-1beta (IL-1β), interleukin-4 (IL-4), interleukin-6 (IL-6), interleukin-10 (IL-10), tumor necrosis factor-alpha (TNF-*α*), interferon-gamma (IFN-*γ*), and transforming growth factor-beta (TGF-β) in serum were determined using enzyme-linked immunosorbent assay (ELISA) kits according to the manufacturer’s instructions (Nanjing Jiancheng Bioengineering Institute, Nanjing, China). All samples were measured in quadruplicate.

#### 2.5.4 Serum antioxidant capacity

The total antioxidant capacity (T-AOC), content of malondialdehyde (MDA), and the activities of superoxide dismutase (SOD), catalase (CAT), glutathione S-transferase (GST) and glutathione peroxidase (GSH-Px) in serum were measured by enzyme labeling instrument (SMR60047, USCNK, China) or spectrophotometer (721G, INESA, China) using commercially available colorimetric diagnostic kits according to the manufacturer’s protocol (Nanjing Jiancheng Bioengineering Institute, China).

### 2.6 Muscle sample collection and analysis

Immediately following exsanguination, the *longissimus dorsi* (LD) muscle samples (about 20 g) over the 12th–13th rib on the left side of the carcasses were collected within 15 min. Muscles were quickly cut into small pieces, stored in a frozen tube (RNase/DNase free) immediately, snap frozen in liquid nitrogen, and stored at −80 °C until RT-qPCR analysis of genes associated with muscle development and fat deposition. The LD muscle samples between 3th and 4th, 6th and 7th, 12th and 13th ribs on the right side of carcasses were collected to evaluate meat quality (pH value, meat color, marbling score, shear force, cooking loss) and chemical composition analysis (moisture, ash, protein, fat).

#### 2.6.1 Assessment of meat quality

The pH of LD muscles was measured at 45 min and 24 h after slaughter by a portable pH meter (Testo 205, Testo Instrument Co., Ltd., Germany). The L^*^ (lightness), a^*^ (redness), and b^*^ (yellowness) of the LD muscles were recorded at 45 min after slaughter by the NR10QC colorimeter (Guangdong 3nh Technology Co., Ltd., China). The pH and meat color at three different sites of the LD muscles were measured, and the average values were calculated. Marbling was scored according to the 5-level scoring system compared to the American NPPC colorimetric plate (1991 edition): trace fat (1), a little bit of fat (2), medium fat (3), too much fat (4), super fat (5). 0.5 points are allowed between two levels, and at least three people participated in the marbling scoring for each LD sample. The meat tenderness meter (MAQC-12, Nanjing) was used to measure the tenderness (shear force, N) of sheep LD muscle samples. The determination methods follow the agricultural industry standard of the People’s Republic of China (NY/T 1180-2006 determination of meat tenderness shear force method). In brief, samples were cooked in an 80 °C water bath until the core temperature reached 70 °C. Then, samples were removed from the water bath and cooled to a central temperature of 4 °C in a refrigerator. Subsequently, the meat samples were taken with a circular sampler (Φ1.27 cm) in a direction parallel to the muscle fibers, and the shear force was determined. The muscle samples used for cooking loss were chilled at 4 °C for 24 h. Then, cooked in a water bath at 85 °C for 20 min. The cooking loss (%) was calculated as a percentage of the weight change of the samples compared to the initial weight of the samples.

#### 2.6.2 Chemical composition analysis

The percentage content (w/w) of moisture, ash, protein and fat in LD muscle samples were analyzed according to GB5009.3-2016 National Food Safety Standard Determination of Moisture in Foods, GB5009.4-2016 National Food Safety Standard Determination of Ash in Foods, GB5009.5-2016 National Food Safety Standard Determination of Protein in Foods, and GB5009.6-2016 National Food Safety Standard Determination of fat in Foods, respectively.

### 2.7 RT-qPCR analysis of genes associated with muscle development and fat deposition

The expression levels of genes associated with muscle development, such as fibronectin 1 (FN1), transforming growth factor beta 1 (TGFβ1), myogenic factor 5 (Myf5), myogenic differentiation 1 (MyoD1), myogenin (MyoG), myostatin (MSTN), and fat deposition, such as fatty acid synthase (FAS), peroxisome proliferator activated receptor gamma (PPARγ), fatty acid binding protein 4 (FABP4), acetyl-CoA carboxylase alpha (ACC), diacylglycerol O-acyltransferase 1 (DGAT1), sterol regulatory element binding transcription factor 1 (SREBF1), carnitine palmitoyltransferase 1A (CPT1A), lipoprotein lipase (LPL), hormone-sensitive lipase (HSL), were detected by real-time quantitative reverse transcription PCR (RT-qPCR).

#### 2.7.1 RNA extraction and cDNA synthesis

The total RNA was extracted from LD muscle samples using a commercially available animal tissue total RNA extraction kit (#DP451, Tiangen Biochemical Technology Co., Ltd., Beijing) according to the manufacturer’s protocol. The RNA quantity and purity were determined using an ultraviolet visible spectrophotometer (#UV1800, RUNQEE, Shanghai). Stock cDNA was synthesized from high-quality RNA (260/280 ratio >1.98) using a commercially available cDNA first chain synthesis kit (#KR103, Tiangen Biochemical Technology Co., Ltd., Beijing) according to the manufacturer’s protocol.

#### 2.7.2 Primer design and RT-qPCR

Primers were based on mRNA sequences^1^ and designed using the Primer-Blast program.^2^ Gene-specific primer sequences are listed in Table 2. RT-qPCR reaction system (total volume 10 μL) consists of 5 μL 2 × SG Green qPCR Mix, 0.2 μL forward primer (10 μM), 0.2 μL reverse primer (10 μM), 1 μL cDNA, and 3.6 μL RNase-free water. cDNA was amplified in triplicate using a QuantStudio^™^ 7 Flex Real-Time qPCR system (ABI, United States). The RT-qPCR reaction procedure includes the following steps: Initial denaturation at 95 °C for 2 min; 40 cycles with denaturing at 95 °C for 5 s, annealing and extension at 60 °C for 30 s. Melting curve analysis was performed by RT-qPCR after 40 cycles of amplification. Data were analyzed using 2^−ΔΔCT^ method.

**Table 2:** List of primer sequences for RT-qPCR.

Gene	Gene ID	Forward primer	Reverse primer	Product length
FN1	100,216,462	CACGTGCTATGACGATGGGA	CTGGTTGTAGGAGTGGGCAG	178
TGFβ1	443,417	CCTGTACAACCAGCACAACC	AGGAGCGCACGATCATGTTG	137
Myf5	443,159	GAGTTCGGGGACGAGTTTGA	CCTCTGGTTAGGGTTGGTCG	273
MyoD1	443,405	TGCACGTCTAGCAACCCAAA	GCTGTAGTCCATCATGCCGT	240
MyoG	443,158	GAGCGTGATCTCCGCTACC	TTGTGGGCATCTGTAGGGTC	164
MSTN	281,187	CAACTTTTGCCCAAGGCTCC	ACTCCGTGGGCATGGTAATG	139
FAS	100,170,327	CTTAACAGCACGTCCCCCAT	TCCTCGGGCTTGTCTTGTTC	149
PPARγ	443,513	GATCTCCAGCGACATCGACC	CTCGCCTTTGCTTTGGTCAG	114
FABP4	100,137,067	GTCCTTCAAATTGGGCCAGG	GGTGGTTGATTTCCCATCCCA	124
ACC	443,186	AAAAAGTACAGGCGGAGCGA	AGATGCTATTCCGCAGGCTC	90
DGAT1	100,126,245	GGTCCGGGACACAGACAAG	AGTTGCTGAAGCCACTGTCA	113
SREBF1	100,329,218	GCTCGTCTTCCTCTGTCTCTCC	GTGGTTGATGCTGGTGGTGTC	94
CPT1A	443,434	TCACATCCAGGCGGCAAGAG	AGCAGAGCGGAATCGTAGACC	119
LPL	443,408	AGACTCCAACGTCATCGTGG	CTCGAAGTTAGGTCCAGCCG	259
HSL	100,169,699	AACAGCAGCGACACAACAGAC	CTCAGACACTTCAGATTCATCCTCAG	120

### 2.8 Rumen fluid sample collection and metabolomic analysis

At the end of feeding experiment, one sheep in each replicate was randomly selected and euthanized, followed by bloodletting and slaughter. Rumen fluid samples (20 mL) were collected in 50 mL anticoagulant-free vacutainer tubes, quickly cooled in liquid nitrogen, and immediately stored at −80 °C until metabolomic analysis.

For metabolomic profiling, weighed samples were homogenized in 400 μL of an internal standard-containing methanol–water solution within 2 mL centrifuge tubes. The extraction protocol involved ultrasonic treatment, incubation, and centrifugation, after which the clarified supernatants were harvested for analysis. A pooled quality control (QC) sample, created by combining equal volumes of all samples, was analyzed alongside the experimental samples to monitor instrument performance. These extracts were analyzed using a Thermo UHPLC-Q Exactive HF-X Mass Spectrometer equipped with an ESI source. The instrument operated in both positive and negative ion modes with a source temperature of 425 °C, sheath/aux gas flows of 50/13 arb, and a spray voltage of 3,500 V. Data were acquired in DDA mode from m/z 70–1,050, using a stepped collision energy (20–40–60 V) for fragmentation. The resolution was set to 60,000 for MS1 scans and 7,500 for MS2 scans. The analysis of metabolomic LC/MS data commenced with preprocessing in Progenesis QI (v4.3, Waters Corp.). The software generated a three-dimensional CSV matrix of sample information, metabolite names, and spectral intensities. A data cleaning pipeline was applied to filter out peaks from internal standards, false positives, and redundant signals. To correct for instrumental and handling variations, we normalized the dataset by the total sum of intensities, followed by a log10 transformation to stabilize variance. For metabolite identification, features were cross-referenced with the HMDB, Metlin, and Majorbio databases.

### 2.9 Statistical analysis

Data analysis was performed by using SPSS 25.0 (SPSS Inc., Chicago, IL, United States). Statistical analysis of growth performance was performed using one-way ANOVA followed by Fisher’s protected least significant difference test, with individual sheep (*n* = 5) as the experimental unit. For all other data, a Student’s *t*-test was performed. All data are expressed as the mean ± standard error of the mean (SEM). Levels for significant differences were set at *p* < 0.05.

The curated data matrix from metabolomic LC/MS was subjected to further analysis on the Majorbio cloud platform. An initial exploratory assessment was performed via principal component analysis (PCA) with the R package “ropls.” To explore the biological context, significant metabolic alterations were investigated through pathway enrichment analysis against the KEGG (Kyoto Encyclopedia of Genes and Genomes) database.

## 3 Results

### 3.1 Diet supplemented with 20% fermented onion improves the growth performance of Liangshan black sheep

As shown in Table 3, compared with the Con group, starting at the same initial weight, adding 20% fermented onion to the basal diet (Trt2 group) significantly increased the final body weight, ADG, and FCR of Liangshan black sheep (*p* < 0.05), while the ADFI in the Trt2 group significantly decreased (*p* < 0.05). On the other hand, adding 10% fermented onion to basal diet (Trt1 group) only significantly increased the ADG of Liangshan black sheep (*p* < 0.05), and adding 30% fermented onion to basal diet (Trt3 group) significantly reduced the final weight, ADG, ADFI and FCR (*p* < 0.05) of Liangshan black sheep (*p* < 0.05). Thus, the basal diet supplemented with 20% fermented onion improves the growth performance of Liangshan black sheep, while the effect of 10% fermented onion is minimal, and even the effect of the 30% fermented onion is negative. Based on the growth performance data, the Con group and Trt2 group were subsequently selected for the following study. The Trt1 and Trt3 groups were not analyzed for other parameters.

**Table 3:** Effect of diet supplemented with fermented onion on growth performance of Liangshan black sheep (*n* = 5).

Items	Group (fermented onion level, %)			
	Con (0)	Trt1 (10%)	Trt2 (20%)	Trt3 (30%)
Initial weight (kg)	23.54 ± 0.57	23.36 ± 0.38	23.40 ± 0.61	23.28 ± 0.33
Final weight (kg)	30.94 ± 0.51^b^	31.52 ± 0.56^ab^	32.10 ± 0.60^a^	28.44 ± 0.62^c^
ADG (g/d/sheep)	211.43 ± 4.85^c^	233.14 ± 5.93^b^	248.57 ± 5.72^a^	147.43 ± 14.65^d^
ADFI (kg/d/sheep)	1.81 ± 0.05^a^	1.86 ± 0.17^a^	1.62 ± 0.09^b^	1.66 ± 0.08^b^
FCR (g/g)	8.55 ± 0.22^b^	8.00 ± 0.86^b^	6.52 ± 0.39^c^	11.37 ± 1.57^a^

The superscripts “a, b, c” indicate that, within each row, values with different superscript letters differ significantly (*p* < 0.05), whereas values with no letters or the same superscript letters are not significantly different (*p* > 0.05).

### 3.2 Diet supplemented with 20% fermented onion improves the health of Liangshan black sheep

As shown in Table 4, all the serum biochemical parameters of the sheep in the two groups were within the normal range. Although there was no significant difference between the experimental group and the control group in TP, ALB, and GLB (*p* > 0.05), the values are slightly higher in the experimental group. Meanwhile, the GPT and GOT in the experimental group sheep were significantly higher than those of the control group (*p* < 0.05), indicating that the sheep in the experimental group had stronger immunity and metabolism.

**Table 4:** Effect of diet supplemented with 20% fermented onion on serum biochemical parameters of Liangshan black sheep (*n* = 5).

Parameters	Con	Trt2	Reference range
TP (g/L)	59.80 ± 6.52	67.21 ± 5.76	60.00–85.00
ALB (g/L)	23.40 ± 2.91	26.04 ± 0.67	20.00–55.00
GLB (g/L)	36.40 ± 4.78	41.17 ± 5.89	20.00–45.00
GPT (U/L)	23.25 ± 0.69	25.82 ± 2.34^*^	0.50–47.00
GOT (U/L)	103.01 ± 2.57^*^	106.93 ± 2.59^*^	12.00–122.00

The superscript “*” means *p* < 0.05 compared with the Con group.

As shown in Table 5, the levels of serum IgA, IgG, IgM, C3, and C4 were significantly increased in sheep fed with a diet supplemented with 20% fermented onion than in those fed with the control diet (*p* < 0.05).

**Table 5:** Effect of diet supplemented with 20% fermented onion on serum immunological parameters of Liangshan black sheep (*n* = 5).

Parameters	Con	Trt2
IgA (μg/mL)	27.17 ± 1.13	35.02 ± 0.36^*^
IgG (μg/mL)	1416.06 ± 31.43	1806.82 ± 34.85^*^
IgM (μg/mL)	13.74 ± 0.44	16.40 ± 0.14^*^
C3 (μg/mL)	595.34 ± 16.96	721.02 ± 10.20^*^
C4 (μg/mL)	241.74 ± 4.12	282.06 ± 9.96^*^

The superscript “*” means *p* < 0.05 compared with the Con group.

As shown in Table 6, the levels of serum IL-1*β*, IL-6, TNF-*α*, and IFN-*γ* were significantly decreased, and the levels of serum IL-4, IL-10, and TGF-β were significantly increased in sheep fed with the fermented onion-supplemented diet (*p* < 0.05).

**Table 6:** Effect of diet supplemented with 20% fermented onion on serum inflammatory factors of Liangshan black sheep (*n* = 5).

Items	Con	Trt2
IL-1β (ng/L)	150.02 ± 5.07	128.07 ± 3.90^*^
IL-4 (ng/L)	107.88 ± 3.22	151.72 ± 2.91^*^
IL-6 (ng/L)	229.28 ± 8.60	182.26 ± 7.39^*^
IL-10 (ng/L)	199.64 ± 6.48	262.83 ± 11.43^*^
TNF-α (ng/L)	1101.20 ± 29.30	933.39 ± 24.02^*^
IFN-γ (ng/L)	66.59 ± 1.33	51.16 ± 0.91^*^
TGF-β (ng/L)	377.68 ± 13.54	443.75 ± 13.78^*^

The superscript “*” means *p* < 0.05 compared with the Con group.

As shown in Table 7, compared with the control diet, the diet supplemented with 20% fermented onion decreased MDA concentration (*p* < 0.05) but increased SOD, GST, CAT, and GSH-Px activity (*p* < 0.05), and the T-AOC capacity in the serum (*p* < 0.05).

**Table 7:** Effect of diet supplemented with 20% fermented onion on serum antioxidant capacity of Liangshan black sheep (*n* = 5).

Parameters	Con	Trt2
MDA (nmol/mL)	7.17 ± 1.29	4.83 ± 0.85^*^
SOD (U/mL)	62.73 ± 3.76	71.35 ± 3.94^*^
GST (U/mL)	58.03 ± 7.31	74.98 ± 11.72^*^
CAT (U/mL)	1.78 ± 0.09	2.15 ± 0.18^*^
GSH-Px (U/mL)	120.00 ± 22.06	240.86 ± 25.25^*^
T-AOC (U/mL)	2.59 ± 0.20	3.21 ± 0.20^*^

The superscript “*” means *p* < 0.05 compared with the Con group.

### 3.3 Diet supplemented with 20% fermented onion improves the meat quality of Liangshan black sheep

As shown in Table 8, compared with the Con group, diet supplemented with 20% fermented onion decreased meat pH_45min_ and pH_24h_ of Liangshan black sheep (*p* < 0.05). In addition, the lightness and yellowness in the Trt2 group significantly decreased (*p* < 0.05), while the redness was significantly increased (*p* < 0.05). The LD muscle in the Trt2 group had a higher marbling score and lower shear force (*p* < 0.05). Meanwhile, compared with the Con group, the diet supplemented with 20% fermented onion also significantly increased the contents of protein and fat in the LD muscle.

**Table 8:** Effect of diet supplemented with 20% fermented onion on meat quality of Liangshan black sheep (*n* = 5).

Parameters	Con (0)	Trt2 (20%)
pH_45min_	6.31 ± 0.01	6.27 ± 0.02^*^
pH_24h_	5.92 ± 0.03	5.87 ± 0.03^*^
Lightness (L^*^)_45min_	30.94 ± 0.23	30.48 ± 0.25^*^
Redness (a^*^)_45min_	16.67 ± 0.37	17.86 ± 0.98^*^
Yellowness (b^*^) _45min_	2.51 ± 0.26	2.09 ± 0.29^*^
Marbling score	2.2 ± 0.27	2.7 ± 0.27^*^
Shear force (N)	66.80 ± 0.97	65.33 ± 0.59^*^
Cooking loss (%)	36.30 ± 1.58	35.88 ± 0.32
Chemical compositions (%)		
Moisture	73.14 ± 0.24	72.91 ± 0.15
Ash	1.27 ± 0.08	1.26 ± 0.06
Protein	21.88 ± 0.37	23.14 ± 0.71^*^
Fat	2.33 ± 0.34	3.32 ± 0.72^*^

The superscript “*” means *p* < 0.05 compared with the Con group.

### 3.4 Effect of diet supplemented with 20% fermented onion on the expression of genes related to muscle development and fat deposition

**Figure 1:**
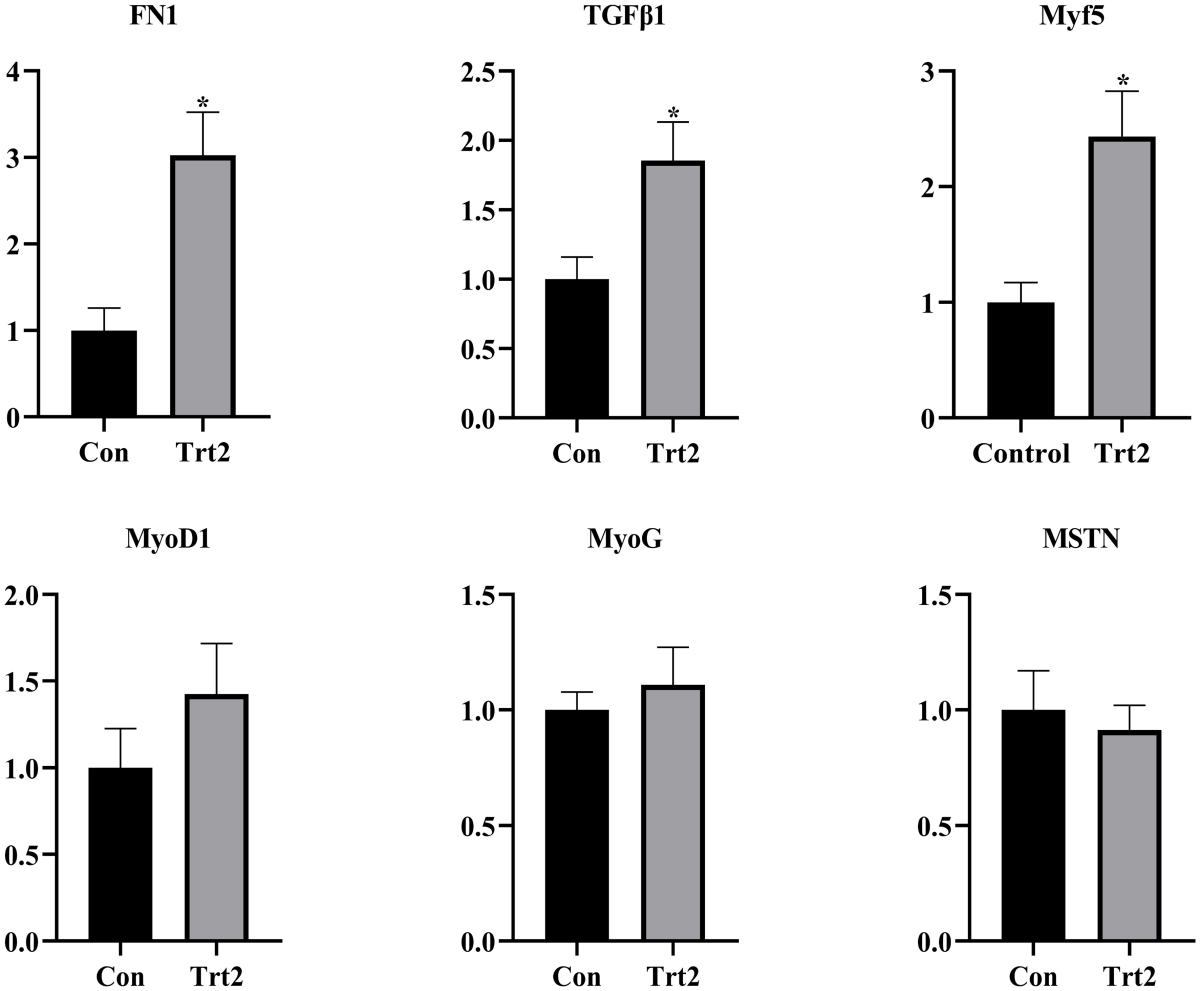
Effect of diet supplemented with 20% fermented onion on the expression of genes related to muscle development. The superscript “*” means *p* < 0.05 compared with the Con group.

As shown in Figure 1, compared with the Con group, the expression levels of FN1, TGFβ1, and Myf5 were significantly increased (*p* < 0.05), the levels of MyoD1 and MyoG showed an upward trend (*p* > 0.05), and the level of MSTN showed a downward trend in the Trt2 group.

**Figure 2:**
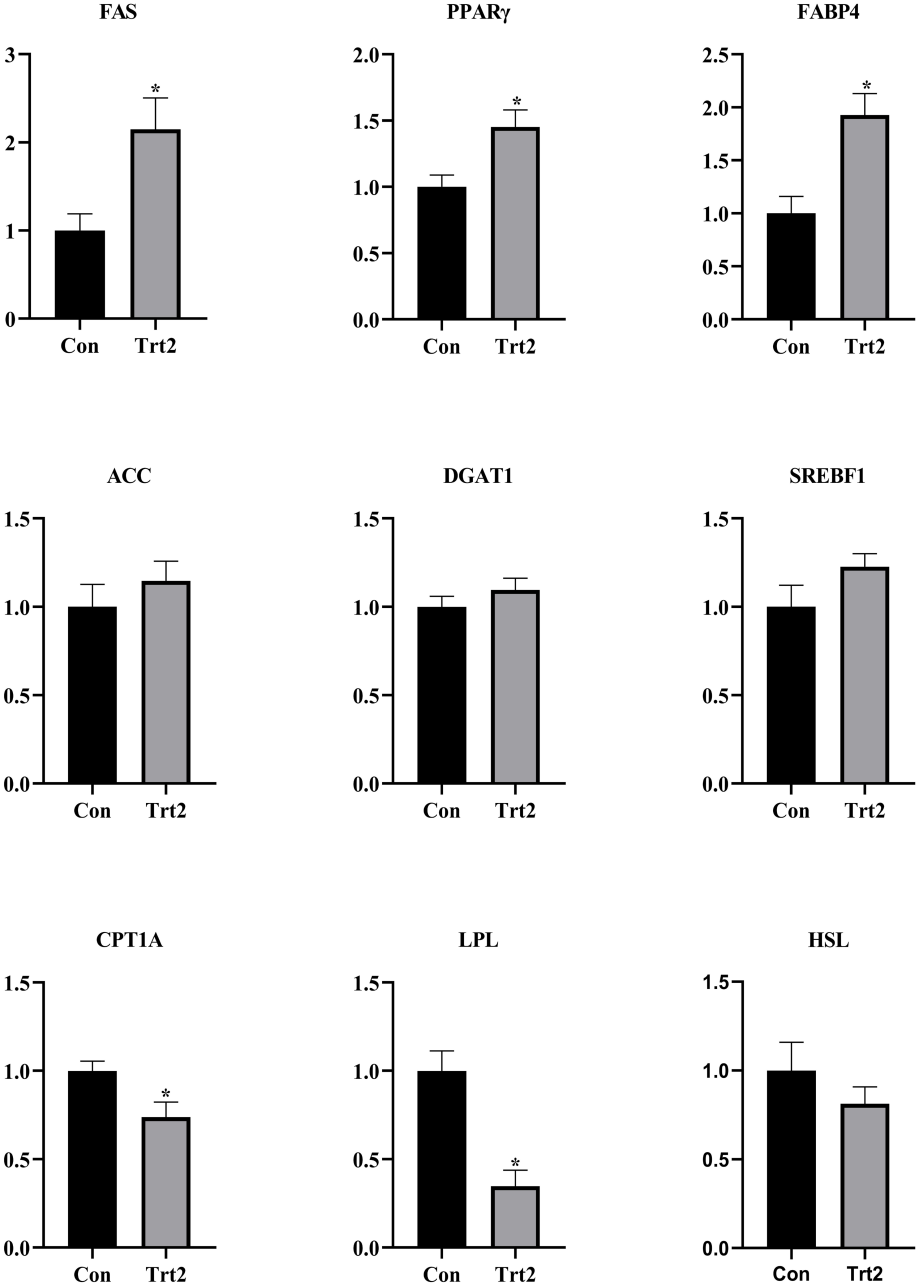
Effect of diets supplemented with 20% fermented onion on the expression of genes related to fat deposition. The superscript “*” means *p* < 0.05 compared with the Con group.

As shown in Figure 2, compared with the Con group, the expression levels of FAS, PPARγ, and FABP4 were significantly increased (*p* < 0.05), and the levels of ACC, DGAT1, and SREBF1 showed an upward trend (*p* > 0.05). In contrast, the levels of CPT1A and LPL were significantly decreased (*p* < 0.05), and the level of HSL showed a downward trend in the Trt2 group.

### 3.5 Effect of diet supplemented with 20% fermented onion on the rumen metabolite profiles of Liangshan black sheep

After the preprocessing steps, peaks with CV >30% in the QCs or present in <80% of the samples were excluded. As a result, 1,143 metabolites were identified in the samples.

**Figure 3:**
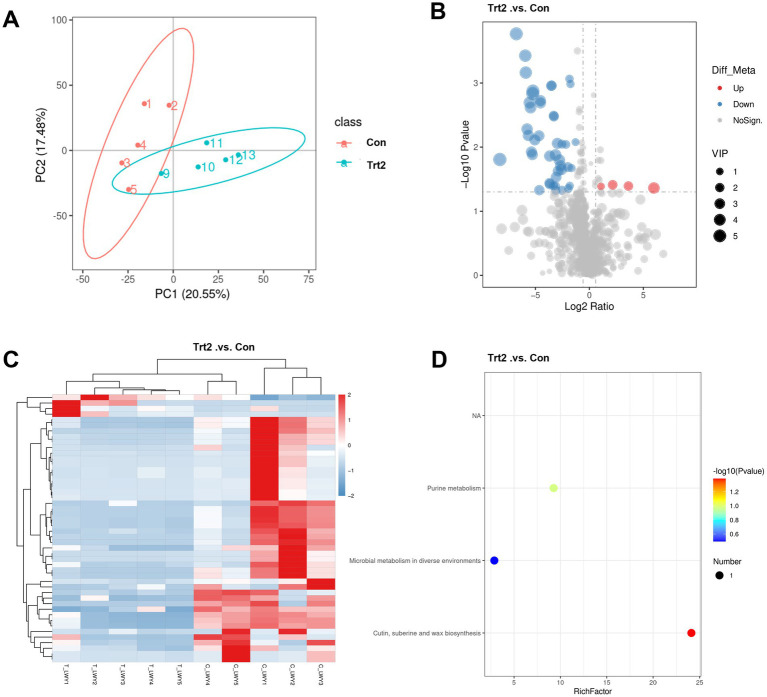
Effect of diets supplemented with 20% fermented onion on the rumen metabolite profiles in Liangshan black sheep. **(A)** PCA, **(B)** volcano analysis, **(C)** heatmap, and **(D)** KEGG pathway enrichment diagrams based on the metabolomic LC/MS data from the rumen liquid of the Liangshan black sheep with (Trt2 group) or without (Con group) 20% fermented onion supplementation.

The PCA score plots (Figure 3A) illustrate the metabolite profiles of rumen fluid from the Con and Trt2 groups. The variance contribution, as indicated by the *R*-squared (*R*^2^) value, represents the extent to which group factors account for the differences among samples. The metabolite profiles of the Trt2 group significantly differ from those of the control group (Con), with PC1 (20.55%) and PC2 (17.48%) as the first and second principal component contributions for these differences, respectively.

The overall distribution of metabolites with significant changes between the Con and Trt2 groups was depicted using a volcano plot (Figure 3B). In this plot, points were color-coded to denote their status: red for significant upregulation, blue for significant downregulation, and gray for non-significance. The contribution of each metabolite, as measured by its VIP value, is represented by the size of the corresponding point. It can be viewed that 44 metabolites were significantly downregulated and four were upregulated by the 20% fermented onion supplementation.

Furthermore, a cluster analysis of the differential metabolites was visualized using a heatmap (Figure 3C). In this visualization, the vertical axis represents the clustered metabolites, the horizontal axis displays the individual samples, and the color scale indicates the relative metabolite abundance. Consistent with the results from Figure 3B, the metabolite information from each group clusters well, in which a large portion of differential metabolites in the Trt2 group shows significant downregulation.

To elucidate the biological functions of the differential metabolites, we performed pathway analysis using the Kyoto Encyclopedia of Genes and Genomes (KEGG) database. The compound names of the differential metabolites, identified in both positive and negative ion modes, were mapped to the KEGG database to retrieve their associated pathways. Subsequently, pathway enrichment analysis was conducted based on a hypergeometric test to determine whether these metabolites were significantly enriched in specific pathways. A *p*-value was calculated for each pathway to assess the significance of the enrichment. As shown in Figure 3D, the results are visualized as a bubble plot, where purine metabolism, microbial metabolism, cutin, suberine, and wax biosynthesis pathways were identified for these differential metabolites between the Con and Trt2 groups.

## 4 Discussion

It was reported that dietary supplementation with onion extracts improves the growth performance and meat quality of broiler chickens (13). However, to the best of our knowledge, no study has been conducted on the effects of a diet supplemented with fermented onion on sheep’s growth performance and health. One previous study demonstrated that, after fermentation, the content of esters, alcohols, and aldehydes in onions was increased, producing a unique and more popular flavor (14). In addition, fermentation increased the quercetin content in onion and subsequently enhanced the antioxidative effect and anti-inflammatory activities (15–17). The appearance, texture, juiciness, flavor, water-holding capacity (WHC), and nutritional value of meat are key parameters in meat production. Color, pH, cooking loss, drip loss, and shear strength are the main indicators of meat quality (18, 19). In this study, the diet supplemented with 20% fermented onion improved the final weight, ADG, and FCR; decreased the ADFI, pH_45min_, pH_24h_, lightness, yellowness, and shear force; increased the redness, the marbling score, and the content of protein and fat. In summary, the diet supplemented with 20% fermented onion improves growth performance and meat quality of Liangshan black sheep. This is consistent with previous studies in sheep (20). Fermented onion contains a large amount of quercetin, which acts as a potential prebiotic with several health-promoting properties (16, 21), including anti-oxidation (15, 22), anti-inflammatory and immune-regulating effects (17, 23), which may be responsible for promoting sheep growth and improving meat quality. However, the beneficial effects of fermented onion on growth performance and the underlying mechanisms warrant further investigation.

The serum biochemical parameters reflect the health condition of animals and the metabolic status of nutrients. The TP content reflects the intake of protein and the level of absorption and utilization of protein. The increase in TP content is beneficial to promote the growth and development of animals and improve the FCR. ALB is a nutrient carrier that maintains plasma osmotic pressure, repairs tissue, and provides energy (24). GLB includes immunoglobulins such as IgA, IgG, and IgM, and complements such as C3, C4, with high content and defensive function, which can enhance the body’s resistance and prevent infection (25). GPT and GOT are important indicators for evaluating liver health. The increase of these two transaminases within a healthy and normal range indicates that the liver has a higher metabolic capacity (26). In this study, it was found that the contents of TP, ALB, and GLB have an increasing trend, and GPT and GOT activities were significantly increased in the Trt2 group. It is likely that the bioactive substances in fermented onion, such as polysaccharides, mercaptan, polyphenols, and flavonoids, could promote the synthesis and metabolism of protein, stimulate the proliferation and differentiation of lymphocytes, and then enhance the immune and metabolism functions (27, 28).

Serum immunoglobulins (IgA, IgM, and IgG) and complement C3 Complement C4 are important components of the humoral immune system and play critical roles in immune responses and gut epithelial protection against pathogens (29). Excessive immunoglobulins stimulate complement components to enhance specific immune mechanisms in birds and protect them against infection (30). In the present study, the levels of IgA, IgM, IgG, C3, and C4 were increased in the serum of Liangshan black sheep fed with diet supplemented with fermented onion.

Inflammatory cytokines play essential roles in immune responses (31). Studies demonstrated that onion polysaccharides have antioxidant, immunomodulatory, antimicrobial, and anti-inflammatory (32–34). Flavonoids extracted from onion inhibited the release of pro-inflammatory factors (e.g., IL-1*β*, IL-6, TNF-*α*, IFN-*γ*) and reduced inflammation (17, 35). IL-4 is an anti-inflammatory factor that inhibits the synthesis and secretion of pro-inflammatory factors such as IL-1, IL-6, and TNF-α. So, IL-4 plays an impartment role in inhibiting the inflammatory response (36, 37). IL-10 is a pleiotropic cytokine with anti-inflammatory properties and inhibits the expression of inflammatory cytokines such as TNF-α, IL-6, and IL-1 by activating macrophages. IL-10 can also inhibit IFN-γ produced by NK cells (38). TGF-β is mainly produced by activated T cells; it inhibits macrophage activation and exhibits anti-inflammatory activity (39). In this study, sheep fed with diet supplemented with fermented onion had low serum concentrations of pro-inflammatory factors such as IL-1β, IL-6, TNF-α, IFN-γ, and high serum concentrations of anti-inflammatory factors such as IL-4, IL-10, and TGF-β. Similar findings are reported by Elattar et al. (17), who demonstrate that onion extracts have promising anti-inflammatory potentials and can effectively suppress the upregulation of pro-inflammatory markers in LPS-stimulated WBCs. Our results suggest that the inclusion of fermented onion in diets enhances humoral immune responses in Liangshan black sheep.

Excessive reactive oxygen species (ROS) may disrupt metabolism in animals, damage cell structures, and accelerate oxidation, thereby causing various diseases (19, 40). SOD, GST, CAT, GSH-Px, and T-AOC are the primary parameters used to evaluate antioxidant levels in organisms. SOD participates in oxygen metabolism, scavenging, and the reduction of superoxide to water and molecular oxygen (19, 41). GST is involved in antioxidant processes that protect cells from free radical damage (42). CAT is a key antioxidant enzyme in living organisms that catalyzes the decomposition of toxic hydrogen peroxide (H₂O₂) into harmless water and oxygen, protecting cells from oxidative damage (42). GSH-Px reduces lipid hydroperoxides to alcohols and free hydrogen peroxide to water (43). T-AOC refers to the total antioxidant level composed of various antioxidant substances and antioxidant enzymes. MDA levels indicate the degree of organic lipid peroxidation and are associated with cell damage (44). Our current study revealed that dietary supplementation with 20% fermented onion increased SOD, GST, CAT, GSH-Px activities and total antioxidant capacity, meanwhile, reduced the concentration of MDA in the serum of Liangshan black sheep. Many studies suggest that onions are rich in quercetin, a strong antioxidant flavonoid that can decrease lipid peroxidation, MDA, but enhance antioxidants such as SOD, CAT, GSH, GPx, and GST activities (33, 45, 46). The findings of our study suggest that fermented onion may be used as a natural antioxidant in sheep and that their antioxidant activity may correlate with growth performance.

FN1 is a macromolecular structural glycoprotein that mediates various cell-extracellular matrix interactions and can promote cell adhesion, migration, growth, and differentiation (47). TGF-*β* can promote cell proliferation, cell differentiation, embryonic development, and tissue fibrosis (48). Myf5, MyoD, and MyoG all belong to myogenic regulatory factors. They participate in muscle development and promote the proliferation and differentiation of myoblasts (49). Conversely, MSTN is a protein that negatively regulates the growth of skeletal muscle, and inactivation of MSTN improves the mass of skeletal muscle (50, 51). In our study, dietary supplementation with 20% fermented onion increased the expression of FN1, TGFβ1, Myf5, MyoD, and MyoG but decreased the expression of MSTN. In summary, dietary supplemented with 20% fermented onion can promote muscle development. This might be one of the reasons why the growth performance and protein content of the sheep in the Trt2 group were higher.

FAS catalyzes the synthesis of fatty acids from acetyl-CoA and malonyl-CoA, thereby playing an important role in body fat deposition (52). PPARγ promotes the differentiation of adipocyte precursor cells into mature adipocytes and participates in lipid storage (53). FABP4 participates in the transportation, storage, and metabolism of fatty acids as well as facilitates the absorption and utilization of water-insoluble dietary long-chain fatty acids (54, 55). ACC is a rate-limiting enzyme that plays a key role in fatty acid synthesis, which catalysis the first committed step of fatty acid synthesis (56). DGAT1 transfers the acyl group from fatty acyl-Coenzyme A to diacylglycerol to synthesize triacylglycerol. It is the key rate-limiting enzyme that controls the synthesis rate of triacylglycerol (57). Triacylglycerol is an important component of intramuscular fat and the most significant form of energy storage in eukaryotic cells (58). Intramuscular fat, which was also subjectively evaluated as a marbling score, represents an important meat quality trait (59). Relatively higher intramuscular fat improves tenderness, making the meat more tender and easier to chew. Intramuscular fat can also improve the water-holding capacity, reduce the cooking loss, maintain the juiciness, and enhance the meat flavor. There are two transcripts of SREBF1 known as SREBP-1a and SREBP-1c. SREBP-1a, which regulates the biosynthesis of cholesterol and fatty acid, exhibits the highest transcriptional activity in the splenic organ and intestinal tract. SREBP-1c is highly expressed in the liver, adipose tissue and skeletal muscle, mainly regulating fatty acid synthesis and participating in the differentiation of adipocytes (60). CPT1 is the rate-limiting enzyme of fatty acid *β*-oxidation. CPT1 contains three isoforms: CPT1A, CPT1B, and CPT1C, and they are highly expressed in the liver, muscle, and brain, respectively. CPT1A transports long-chain fatty acids to mitochondria for β-oxidation, thereby providing energy for cells (61, 62). LPL and HSL are two important enzymes involved in lipolysis. LPL decomposes triglyceride into glycerol and free fatty acids (63). Hormone-sensitive lipase (HSL) is responsible for releasing free fatty acids from adipose tissue into circulation (64). In this study, dietary supplementation with 20% fermented onion increases the expression of genes related to fat deposition (FAS, PPARγ, FABP4, ACC, DGAT1, and SREBF1) while reducing the expression of genes related to fat breakdown (CPT1A, LPL, and HSL). This explains the reason why the marbling score and fat content of the mutton in the Trt2 group were higher.

The metabolomic analysis of rumen fluid provides a critical biochemical lens through which to interpret the significant improvements in growth performance observed in sheep supplemented with 20% fermented onion. The profound shift in the rumen metabolite profiles, characterized by the downregulation of 44 metabolites and upregulation of four, strongly suggests that 20% fermented onion acts as a potent modulator of the rumen microbial ecosystem. The enrichment of these differential metabolites in specific pathways, notably purine metabolism, microbial metabolism in diverse environments, and cutin/suberine biosynthesis, offers a compelling mechanistic basis for the enhanced average daily gain (ADG) and final body weight. The downregulation of numerous purine metabolites is particularly intriguing. In the context of the rumen, purines (e.g., adenine, guanine) and their degradation products (e.g., hypoxanthine, xanthine, uric acid) are central to microbial nucleic acid synthesis (65). A decrease in their free concentration in the rumen fluid may not indicate reduced production, but rather a more efficient capture and utilization by the microbial biomass for proliferation. This heightened efficiency in microbial synthesis would lead to an increased yield of microbial crude protein (MCP), which is the primary source of high-quality protein for the host ruminant (66). This proposed increase in MCP flow to the small intestine directly supports the observed improvements in growth performance and aligns with the upregulation of myogenic-related genes such as Myf5.

Furthermore, the significant alterations within the “microbial metabolism in diverse environments” pathway underscore a fundamental reshaping of the rumen’s metabolic network. Fermented onion, rich in prebiotics like fructans, probiotics from the fermentation process, and bioactive organosulfur compounds, likely selects for a more efficient microbial community (67, 68). This could involve a shift from less efficient proteolytic fermentation, which can produce wasteful compounds like ammonia, to more robust saccharolytic fermentation, maximizing the production of volatile fatty acids (VFAs) (69). Although VFAs were not directly measured, they are the primary energy source for ruminants (70), and an enhanced production would directly fuel the energy-demanding processes of growth and maintenance, explaining the increased average daily feed intake (ADFI) as the animals’ metabolic capacity expanded. The enrichment in the “cutin and suberine biosynthesis” pathway, which involves the breakdown of complex plant polymers, points towards an enhanced fibrolytic capability of the rumen microbiota. The downregulation of certain intermediates in this pathway could signify a more complete and efficient degradation of the basal diet’s fibrous components, unlocking previously inaccessible energy and nutrients. Collectively, these metabolomic shifts paint a coherent picture of a rumen environment reprogrammed by fermented onion for superior nutrient conversion efficiency, providing both the protein building blocks (via enhanced MCP) and the energy (via enhanced VFA production and fiber digestion) necessary to drive the remarkable improvements in the growth performance of Liangshan black sheep (71).

## 5 Conclusion

This study revealed that diet supplemented with 20% fermented onion improved growth performance, health, and meat quality in Liangshan black sheep through enhancing immunity, anti-inflammatory ability, antioxidant capacity, and modifying the rumen metabolite profiles.

## Data Availability

The raw data supporting the conclusions of this article will be made available by the authors, without undue reservation.

## Footnotes

^1^ http://www.ncbi.nlm.nih.gov/nucleotide

^2^ http://www.ncbi.nlm.nih.gov/tools/primer-blast/
